# Pulmonary embolism with abdominal pain as the chief complaint: A case report and literature review

**DOI:** 10.1097/MD.0000000000017791

**Published:** 2019-11-01

**Authors:** Yu Han, Yuxin Gong

**Affiliations:** a The Second Clinical Institute, Southern Medical University; b Department of Respiratory, Zhujiang Hospital of Southern Medical University, Guangzhou, China.

**Keywords:** abdominal pain, diagnosis, pulmonary embolism, pulmonary infarct

## Abstract

**Rationale:**

Pulmonary embolism (PE) is one of the serious cardiopulmonary diseases that can endanger life. Early diagnosis and timely treatment are key factors to reduce its high mortality rate. Abdominal pain is not currently included in the symptoms of PE in textbooks and guidelines.

**Patient concerns:**

A 49-year-old man was hospitalized for an exacerbation of right upper quadrant abdominal pain and sudden left upper quadrant pain that lasted for 2 hours.

**Diagnoses:**

The patient was initially misdiagnosed as cholecystitis and pneumonia, and later was diagnosed as PE by computed tomography pulmonary angiography (CTPA).

**Interventions:**

The patient received low molecular weight heparin for anticoagulant therapy.

**Outcomes:**

His abdominal pain disappeared after one week. The patient was later discharged.

**Lessons:**

Sometimes abdominal pain may be the only manifestation of PE. However, most clinicians do not think of the possibility of PE in patients with abdominal pain. This might have contributed greatly to the rate of misdiagnosis of PE in the past. We hope to improve the alertness of the diagnosis of PE in clinical practice. In patients with abdominal pain, the possibility of PE should be considered to avoid mis- or under-diagnosis.

## 1 Introduction

Pulmonary embolism (PE), one of the major contributors to the global disease burden, is associated with significant morbidity and mortality. The diversity and low specificity of clinical manifestation of PE results in frequent misdiagnosis or missed diagnosis. It is reported that the missed diagnosis rate is more than 70%, and only 7% of PE patients who die are diagnosed before their death.^[1]^ In contrast to the hospitalization mortality rate of PE ranging from 2.5% to 10%,^[2]^ the mortality rate of missed diagnosis PE patients is generally considered to be 30%,^[1]^ indicating a catastrophic harm of misdiagnosis or missed diagnosis of PE. Correct early diagnosis and timely treatment are therefore key factors to reduce the mortality rate of PE.

Until now, all textbooks, authoritative reviews and guidelines on PE describe its symptoms as dyspnea, chest pain, cough, hemoptysis, etc.,^[3–5]^ with no mention of abdominal pain. Similarly, PE was not mentioned in the differential diagnosis for acute abdomen in early review studies.^[6,7]^ It seems that abdominal pain has nothing to do with PE, making it difficult for physicians to link the two. However, this cognition needs to be changed.

## 2 Case report

**Figure 1:**
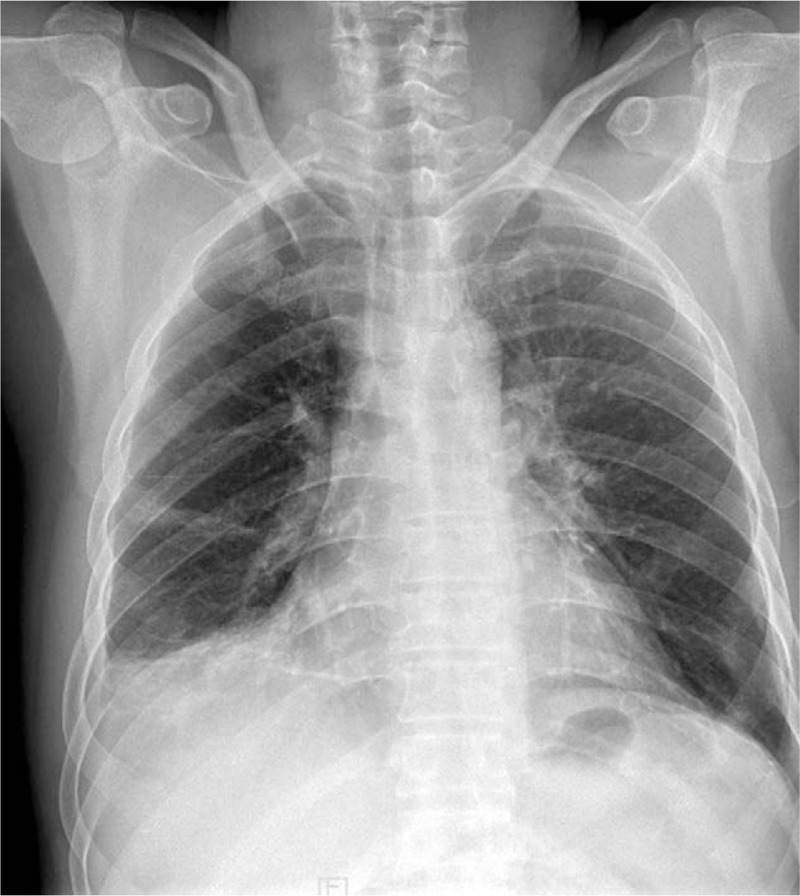
Chest radiograph revealed infiltration shadow in lower part of both lungs.

**Figure 2:**
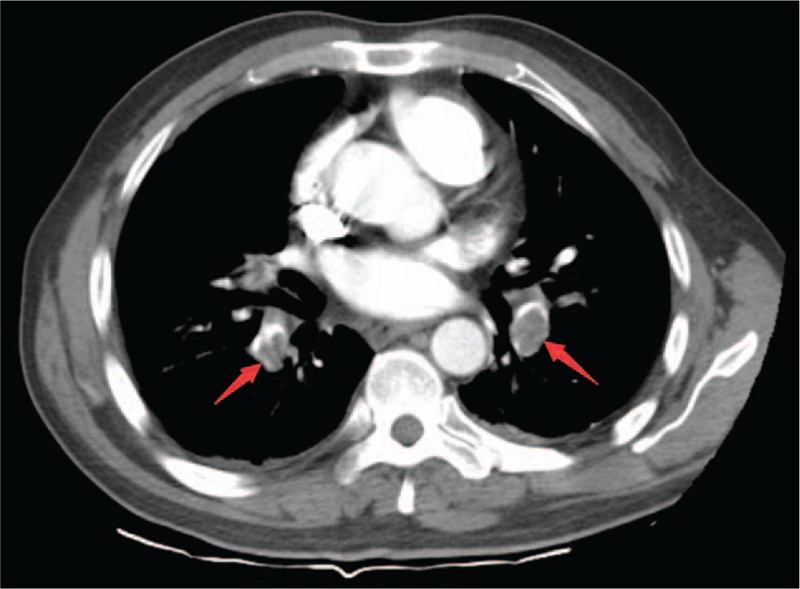
CTPA revealed multiple embolism in bilateral pulmonary artery (arrow). CTPA = computed tomography pulmonary angiography.

**Figure 3:**
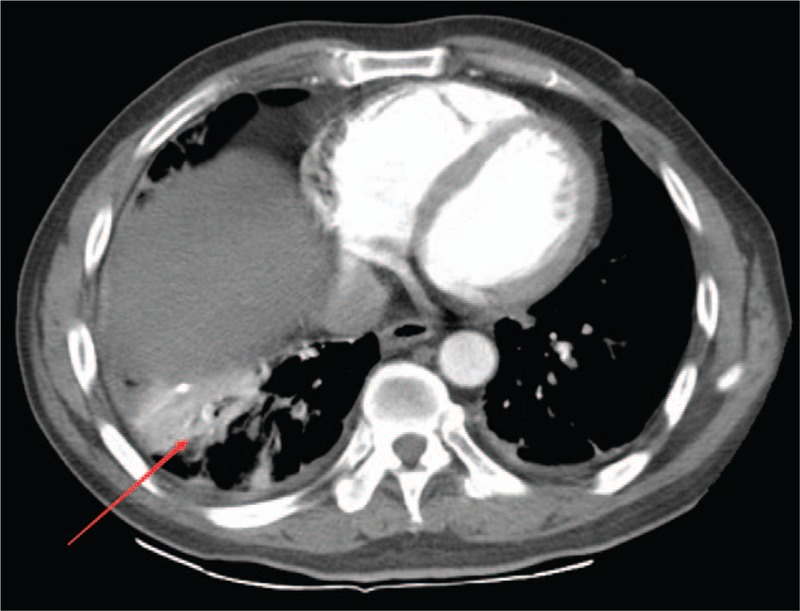
CTPA revealed right lower pulmonary infarctions (arrow) and a small amount of pleural effusions on both sides. CTPA = computed tomography pulmonary angiography.

A 49-year-old man was hospitalized for an exacerbation of right upper quadrant abdominal pain and sudden left upper quadrant pain that lasted for 2 hours. He had developed persistent right upper quadrant pain 10 days ago with axillary temperature at 37.5°C, the local clinic considered cholecystitis and treated him with antibiotics and the low fever disappeared, but the pain did not improve. There were no other symptoms. Cardiopulmonary auscultation and abdominal ultrasonography were both unremarkable. He had no significant medical history. Blood amylase, liver function, and blood routine were all within normal limits. Chest radiograph revealed infiltration shadow in lower part of both lungs and a small amount of pleural effusion on the right side (Fig. 1). With preliminary diagnoses of pneumonia, the patient was given antibiotic treatment. But then PE was suspected when arterial blood gas test revealed that his SpO_2_ was 92% on room air. Plasma D-dimer was 8.8 mg/L. Lower extremity deep venous ultrasound revealed right peroneal vein and calf intramuscular vein thrombosis of the lower leg. Bedside color Doppler echocardiography revealed tricuspid regurgitation 3.7 m/s and pressure difference 56mmHg. Further examination with computed tomography pulmonary angiography (CTPA) revealed multiple embolism in bilateral pulmonary artery trunk, second and third order branches, bilateral pleural effusion and bilateral multiple pulmonary infarction (Figs. 2 and 3). The patient received low molecular weight heparin for anticoagulant therapy. His abdominal pain was significantly relieved after 4 days and disappeared after one week. After discharge, the patient continued to take warfarin orally for anticoagulant treatment.

## 3 Discussion

For abdominal pain, we usually think of abdominal disease first, followed by myocardial infarction or pleurisy, and almost no doctors will think of pulmonary embolism. In this case report, the patient was hospitalized for abdominal pain without the typical symptoms of PE such as chest tightness, shortness of breath and dyspnea. She was initially misdiagnosed as cholecystitis and pneumonia. It was not until the patient's hypoxia was discovered and a series of workups were performed that the correct diagnosis of the PE was finally made, avoiding the serious consequences that may occur.

For this reason, we conducted a literature review on abdominal pain and PE. To our surprise, abdominal pain is reported in about 11% patients with PE, based on a few case studies. The earliest report was in 1957, in which 11 (12.2%) of the 90 cases of PE had abdominal pain, of which 6 were their chief complaint.^[8]^ The most recent report was a large study in 2011, in which 202 (10.7%) of 1880 patients diagnosed with PE had upper abdominal pain.^[9]^ In fact, in the recent 10 years, relevant reviews of acute abdomen have begun to suggest that PE should be considered for upper abdominal pain.^[10,11]^ The 2015 guidelines for acute abdomen in Japan stated that acute PE is one of the super acute diseases that should be excluded in the diagnosis of acute abdomen.^[12]^

We searched similar cases in the PubMed/MEDLINE database using keywords “abdominal pain”, “Pulmonary embolism” and synonyms such as “acute abdomen” and “flank pain”, et al. Through literature search, we identified additional 31 cases of PE with abdominal pain manifestation,^[13–33]^ total 32 cases in combination of our case. All cases of abdominal pain are associated with PE. In 20 cases (62.5%), abdominal pain was the only symptom of PE. Twenty-three cases (71.9%) were initially misdiagnosed/missed. The lower lung or basal lesions were identified in 25 cases (78.1%). Twenty-six (83.9%) out of 31 patients with the statement of location of abdominal pain had superior abdominal pain. Of the 22 cases with unilateral lung lesions, except for 1 case of epigastric pain, the abdominal pain side and the lung lesion side in 21 (95.5%) cases were the same side. The location of abdominal pain in bilateral lung lesions was irregular in 10 cases.

Most PE patients with abdominal pain have no typical symptoms of PE, resulting in a high rate of misdiagnosis. Location of abdominal pain is mostly in the upper abdomen, especially the ipsilateral side of pulmonary lesions. Most cases are lower lobe/basal embolism or infarction. Almost all misdiagnoses point to abdominal diseases. Except for incidental findings, the diagnosis of PE is often based on expanded workups due to ineffective treatment or the inability to determine the disease, or after the typical signs and symptoms become more evident during the disease evolvement. The lack of symptoms of abdominal pain in the traditional perception of PE, as well as the decreased attention paid to prompt information of PE under the guidance of abdominal diseases, are the main reasons for the misdiagnosis.

There are a variety of possible explanations for the mechanism of abdominal pain. Some suggests that PE can lead to abdominal disease: Abdominal pain may be caused by liver capsule or gallbladder dilation secondary to right-sided heart failure-induced by PE.^[8]^ This explanation is believed to be one of the main causes of abdominal pain caused by PE. In an acute abdominal literature, the location of abdominal pain considering the possibility of PE was considered to be limited to the right upper quadrant^[10]^; Increased right ventricle pressure may lead to re-opening of the patent foramen ovale, which may result in paradoxical embolism, causing decreased blood supply to the abdominal organs^[34]^; The increased viscosity of blood with low oxygen may lead to small embolus that causes focal necrosis of abdominal organs^[26,35]^; Pulmonary hypertension may lead to abdominal lymphedema and hepatobiliary portal infiltration^[22]^; PE may lead to neurological disorders,^[8]^ such as pseudoileus^[36]^; Some believes that the abdominal pain is actually referring chest pain: Which may be caused by pulmonary hypertension or thrombus stimulation of sensory nerve endings in the blood vessel wall^[37]^; The pain is caused by lateral stimulation of the diaphragm.^[8]^ Alternatively, the abdominal pain may be caused by the tension of sensory nerve endings within the lower part of the chest wall parietal pleura and intercostal muscle hyperalgesia.^[37]^

Pleurisy can manifest as abdominal pain, and the literature collating the ipsilateral nature of lung lesions and abdominal pain also provides more support for the Thoracogenic explanation. Basilar lung infarction with exudation or bleeding, can lead to stimulation to the pleura via inflammatory reaction and localized fibrous adhesions that may involve the diaphragm and even peritoneum. The stimulated intercostal and diaphragm nerve pain then manifests as the referred abdominal pain.^[31,38]^ We agree with this explanation, based on the analysis of our case.

In as such, abdominal pain is not that rare in PE manifestation, and even presents as the chief complaint. However, most clinicians do not think of the possibility of PE in patients with abdominal pain. This might have contributed greatly to the rate of misdiagnosis of PE in the past, but should not be continually ignored from now on.

In patients with abdominal pain, PE should be considered in the differential diagnosis, especially in those with risk factors such as malignancy, nephrotic syndrome, immobility, diabetes, connective tissue disease, lower extremity deep vein thrombosis, pregnancy, oral hormone and contraceptive pills. Early detection of blood oxygen saturation and D-dimer should be performed, and the presence of PE should be considered in highly suspicion patients.

## Author contributions

**Conceptualization:** Yu Han, Yuxin Gong.

**Data curation:** Yu Han.

**Formal analysis:** Yu Han.

**Investigation:** Yu Han.

**Methodology:** Yu Han, Yuxin Gong.

**Project administration:** Yu Han, Yuxin Gong.

**Resources:** Yu Han.

**Supervision:** Yuxin Gong.

**Validation:** Yu Han, Yuxin Gong.

**Visualization:** Yu Han.

**Writing – original draft:** Yu Han.

**Writing – review & editing:** Yu Han, Yuxin Gong.

Yuxin Gong orcid: 0000-0001-7449-5737.
